# Respiratory Infections in the American Expeditionary Forces

**Published:** 1918-10

**Authors:** Haven Emerson


**The Experience of the American Expeditionary Forces with Respiratory Infections.** Major HAVEN EMERSON, M. R. C.

The speaker said in part :

Respiratory infections are understood to be those diseases which are passed by the moist discharges from the bronchi or trachea, the throat, and the nose and mouth, i. e., the saliva and spray. Of the diseases included in the nomenclature of the sick and wounded reports of the A. E. F., the following eleven diseases are considered respiratory infections for the purpose of this paper : —

| A. | B. |
|---|---|
| Mumps | Bronchitis (acute) |
| Measles | Influenza |
| Scarlet fever | Pneumonia (lobar) |
| Diphtheria | Tonsilitis (acute) |
| Meningitis (meningococcus) | Pneumonia (broncho) |
| | Pharyngitis (acute) |

Rhinitis and laryngitis are not mentioned since they are so rarely included in the sick and wounded reports as having resulted in hospitalization.

Sinusitis, otitis and the various secondary involvements commonly following upper respiratory tract infections are not included because such inclusion would at once take the discussion too far afield and involve us in too many clinical and pathological uncertainties as to origin of infection.

It will be noted that the eleven diseases listed fall naturally into two groups : (A) including diseases commonly recognized and feared as communicable, and against which well considered and logical measures are generally and effectively employed ; (B) representing diseases which people have always considered as afflictions of fate, for which no particular individual is responsible, and against which no well defined measures are worth instituting, or are likely to prove effective. The order in which the two groups are listed above is the order for each group according to the number of days lost from hospitalization of each disease.

Not less than 50 o/o of the diseases in group A are properly attributable to the conditions of overcrowding on the transports from the United States and in camps at Base ports, not less than half of all the cases so far reported occurring at the Base ports or within such period after embarkation in England and France as to prove that the infection must have taken place prior to debarkation or in the holding or " rest camps " at the Bases.

The incidence rate for pneumonia per 100,000 troops parallels so closely the rate of the other diseases under consideration that the rate of this disease can best be used as a basis of comparison between the experience of the British Armies in France and that of the A. E. F.

*Case Rates of Pneumonia per 100,000 Men Strength per Month.*

| A. E. F. | 1917 | B. E. F. | A. E. F. | 1918 | B. E. F. |
|---|---|---|---|---|---|
| | January . . . | 8.67 | 392.3 | January . . . . | 17.16 |
| | February . . . | 25.27 | 155.3 | February . . . | 5.65 |
| | March . . . . | 14.58 | 163.9 | March. . . . . | 10.11 |
| | April. . . . . | 10.40 | 59. | April . . . . . | 11.80 |
| | May . . . . . | 8.93 | 16.5 | May. . . . . . | 7.65 |
| | June. . . . . | 6.23 | 82.2 | June . . . . . | 10.24 |
| 66.6 | July . . . . . | 7.35 | 44.6 | July | |
| 26.9 | August. . . . | 4.68 | 40.7 | August | |
| 20.4 | September . . | 3.49 | | | |
| 70.21 | October . . . | 5.68 | | | |
| 66.0 | November . . | 6.65 | | | |
| 213.3 | December. . . | 7.55 | | | |

With the exception of the incidence of pneumonia among our colored troops and service regiments, which has been about ten times the rate among the white A. E. F. troops, there has been no selective susceptibility of race shown by a study of the figures.

For pneumonia as for the other respiratory infections, here, as in civilian experience at home, crowding and close personal contact and exposure while suffering from lowered resistance, due to a variety of unfavorable conditions of occupation and environment, is apparently the dominant and in many instances the sole determining factor in causing the serious increase of respiratory infections among our troops in France.

Of the 1,613,500 days lost in the A. E. F. up to June 1st, 1918, from all causes — sickness, accident, injury in action and other traumatisms — 1,481,000 days or 91.78 o/o were lost because of sickness.

Of the total loss of days due to hospitalization from sickness 631,226 days or 42.61 o/o were due to the eleven respiratory infections under consideration. The total case incidence of these eleven diseases by months from June 1917 to August 1918 has been as shown in (A), and the rate per 100,000 troops in the A. E. F. as shown in (B).

| 1917 | A. | B. | 1918 | A. | B. |
|---|---|---|---|---|---|
| June | 31 | 221 | January | 16,843 | 8,637 |
| July | 752 | 5,013 | February | 14,412 | 6,185 |
| August | 913 | 3,126 | March | 10,135 | 3,519 |
| September | 1,203 | 2,455 | April | 6,145 | 1,934 |
| October | 2,798 | 3,781 | May | 3,102 | 571 |
| November | 6,925 | 6,528 | June | 2,999 | 1,708 |
| December | 12,749 | 8,499 | July | 11,096 | 1,131 |
| | | | August | 12,457 | 969 |

The striking effect of the epidemic of influenza coming at a time of year when other respiratory infections are usually at their minimum is shown in the sudden increase in rate in June.

In December 1917 and January 1918 the A. E. F. lost 113,244 days on account of sickness in hospital from these particular diseases, with a mean strength of 172,876 men, or 65 days per capita. With a force of 2,000,000 men and the same incidence of the diseases, we shall need 21,666 beds continuously for these two months to care for these diseases in the coming winter.

Based on the experience of the past 15 months (112,560 cases) we find the average number of days lost for each case of the diseases respectively to be as follows : —

| | |
|---|---|
| Pneumonia (lobar) | 22.7 days. |
| Meningitis (meningococcus) | 20.7 " |
| Scarlet Fever | 14.8 " |
| Mumps | 12.0 " |
| Pneumonia (broncho) | 11.7 " |
| Measles | 10.3 " |
| Diphtheria | 7.2 " |
| Bronchitis (acute) | 6.3 " |
| Pharyngitis (acute) | 4.3 " |
| Tonsilitis (acute) | 4.1 " |
| Influenza | 3.4 " |

The above average of days lost for each disease is based on the total number of cases admitted to hospital and the total days lost from service from each disease.

Among the causes which are known to have contributed to the high incidence of the diseases in question are : —

Crowding in camps while awaiting, or in trains en route for, embarkation at U. S. Ports.

Crowding, inadequate ventilation, insufficient medical inspection, and isolation facilities on transports.

Crowding at ports of embarkation (in one instance the floor space per capita being only 8 sq. ft. in barracks).

Crowding in barracks throughout France, twenty square feet and 200 cubic feet per capita being the official allowance in regulation barracks with double tier bunks (British and French provision except in front areas being 40 sq. feet and 400 cubic feet per capita).

Exhausting drilling beyond the point of reasonable fatigue.

Working and sleeping sometimes in wet clothing and shoes.

Eating of food served cold, and without shelter from cold and wet while eating.

Insufficient blankets for warm sleeping.

Practice of awaiting complaint of sickness by men instead of searching constantly for early cases of infections with coughs, colds, and other early symptons, and segregating them for observation, and if necessary for hospitalization.

Habitual neglect of open window ventilation of barracks at night in cold weather.

Spitting promiscuously in and around barracks, mess halls, kitchens, etc., and failure to protect the nose and mouth when coughing and sneezing among groups of men.

Some of the unfavorable conditions responsible for the high rate of respiratory infections are the inevitable result of the conditions of transportation on sea and land imposed by military operations and the imperative requirements of speed in troop movements.

Some are the result of limitations in labor and materials for the construction of shelter buildings for sleeping and eating.

Some result from lack of imagination on the part of medical officers, whose experience has been chiefly in private practice where the prevention of disease in the individual from spreading to the community, as a group of contacts, has been subordinated to the treatment of the patient who comes with a complaint of symptoms.

Some have been due to lack of discipline, in matters of personal hygiene, of the men in barracks, billets, and tents, owing to the indifference or disregard by line officers of the warnings and advice of medical officers.

Whatever may be the limitations of space or shelter, conveyance or crowding, imposed by the prosecution of the war, there is much that can be done at once by a determined effort to remove every least accessory embarrassment or hindrance to the protection of the enlisted men of the A. E. F. from preventable respiratory infections.

The following plan of action is suggested as capable of adaptation to the needs as they are now found in France. Fix as the minimum floor space per capita for sleeping quarters 40 square feet wherever the billeting or allotment of barrack space can be controlled.

Wherever the number of men ordered to occupy barracks or billets exceeds this ratio the following resources should be used : —

*a.* Thinning out by providing tentage or makeshift outdoor shelter with increase of blanket allowance to those outside of buildings.

*b.* Alternate head to foot arrangement of men in bunks with such supervision as will insure the observance of this order. (The British have found it necessary to fix the bunks to the floor and lower the foot of bunks several inches to insure observance of the head to foot order).

*c.* Where heads are unavoidably at same end of closely adjacent bunks, the arrangement of partition by wood or paper for 2 feet down from head of bunk to prevent spray infection.

Everywhere : —

*a.* Provision for bathing facilities with hot water, drying rooms for shoes and outer clothing, food served hot and under shelter from cold and wet, and boiling water for rinsing cleaned mess kits.

*b.* Policing of barracks regularly at night to insure free continuous flow of fresh air through open windows in all barracks and billets.

*c.* Search for early symptoms and physical signs of catarrhal affections of eyes, nose, throat and chest and taking of temperatures in suspicious cases, with segregation of hospitalization without waiting for well declared cases of infection.

*d.* Removal of common drinking cup from Lyster bags and other water supply points and discontinuance of the habit of sucking water from the up-turned nipple of the Lyster bag.

*e.* Avoidance of drilling tired men especially when infection is prevalent and during periods of severe weather.

Many studies of the epidemiologists indicate that reduction of resistance by fatigue, cold and hunger is as important as contact in causing spread of pneumonia.

Every resource which ingenuity and determination can provide must be used to overcome the handicap of crowding.

The avoidance of severe wastage of man power this winter from respiratory infections is worthy of the best brains and most devoted service of the Medical Staff.

